# Intellectual Disability and Microcephaly With Pontine and Cerebellar Hypoplasia in Adults: Severity Spectrum and Diversity of the CASK Gene Aberrations

**DOI:** 10.7759/cureus.109777

**Published:** 2026-05-27

**Authors:** Koji Obara, Erika Abe, Itaru Toyoshima

**Affiliations:** 1 Neurology, National Hospital Organization Akita National Hospital, Yurihonjo, JPN

**Keywords:** adult, cask, micpch, scoliosis, x chromosome inactivation

## Abstract

Intellectual disability and microcephaly with pontine and cerebellar hypoplasia (MICPCH) is a rare X-linked neurodevelopmental disorder characterized by severe developmental delay, microcephaly, and distinctive brain MRI findings, predominantly affecting females due to loss-of-function variants in the calcium/calmodulin-dependent serine protein kinase (*CASK)* gene. We describe three adult female patients with MICPCH, aged 31, 43, and 50 years, exhibiting core symptoms of severe intellectual disability, postnatal microcephaly, and growth retardation. Patients 1 and 2 failed to achieve standing, had severe scoliosis, and received tube feedings. Patient 3 had mild symptoms, could gain ambulation, had mild scoliosis, and could take food orally. Genetic analyses of the *CASK* gene revealed a heterozygous nonsense variant, c.1609C>T, p.(Arg537*) in patient 1, a novel frameshift deletion, c.1079_1089del, p.(Leu360Glnfs*2) in patient 2, and a copy number variant, a heterozygous duplication of exons 12-14 in patient 3. Our adult cases expand the known age spectrum and phenotypic variability of MICPCH associated with *CASK* aberrations. Despite relatively similar MRI findings, the patients exhibited substantial differences in functional severity, epilepsy burden, and scoliosis progression, highlighting the dissociation between radiological and clinical severity in MICPCH. These findings emphasize the importance of long-term multidisciplinary management in adult patients.

## Introduction

Intellectual disability and microcephaly with pontine and cerebellar hypoplasia (MICPCH: OMIM 300749) is a rare neurodevelopmental disorder and a genetically heterogeneous condition [1-3]. Still, it is typically seen in females and is generally associated with pathogenic loss-of-function variants in the calcium/calmodulin-dependent serine protein kinase (*CASK)* gene [2,4]. The *CASK* gene on the X chromosome at Xp11.4 encodes *CASK*, a member of the membrane-associated guanylate kinase (MAGUK) protein family [3,5]. *CASK* is integral to neural development and synaptic functions, influencing processes ranging from synaptic interactions to dendritic spine formation [4]. MICPCH is characterized by short stature, severe postnatal microcephaly, severe developmental delay /intellectual disability, various types of seizures, motor developmental delay, axial hypotonia with or without hypertonia of extremities, and mild facial dysmorphisms [2,4].

Moreover, brain MRI findings in MICPCH are diagnostic hallmarks, which show pontine and cerebellar hypoplasia with a normal-sized corpus callosum [2,6]. Cerebellar hemispheres are often affected asymmetrically [2,6]. Researchers have reported nearly 200 cases of MICPCH associated with aberrations in the *CASK* gene [7]. Most cases involve children, with only a few reports of adults, the oldest being a 32-year-old patient [8]. Therefore, the clinical spectrum and long-term outcomes in adult patients with MICPCH remain underexplored. Here, we describe three adult cases with MICPCH. The patients were 31-, 43-, and 50-year-old women with a known nonsense variant, a novel frameshift variant, and a copy number variant in the *CASK* gene, respectively.

## Case presentation

Patient 1

Patient 1 was a 43-year-old woman who was born to non-consanguineous parents. Her mother's pregnancy and delivery were normal. Although the birth weight, length, and head circumference were unavailable because the original neonatal records could not be retrieved, her mother recalled that microcephaly had been pointed out during the neonatal period. However, the exact timing, head circumference measurements, and diagnostic criteria could not be confirmed retrospectively. At age three years, she could sit independently and crawl, but she did not achieve further motor milestones such as standing or walking. She could not speak but could make eye contact with caregivers. At age 22 years, she became bedridden, totally dependent on activities of daily living, and gradually lost eye contact.

She developed her first seizure at age three years, characterized by brisk head drops. Thereafter, she continued to experience frequent generalized myoclonic seizures and tonic seizures lasting approximately 15 seconds once or twice daily. Despite treatment with various antiseizure medications, seizure control remained difficult for many years. At age 35 years, perampanel was introduced at 7 mg/day, after which the seizure frequency decreased.

She lived at home until the age of 37, when she developed pneumonia, was hospitalized, underwent gastrostomy and tracheostomy, and was ventilated mechanically. She was then transferred to our institution at age 38. At the present age of 41, her height was 125 cm, body weight was 32.5 kg, and head circumference was 46 cm. She had dysmorphic facial features, including a round face, a broad nasal bridge, and large ears. Further, she showed severe scoliosis and overriding toes. On neurological examination, she was alert but did not utter words or follow instructions. She did not show nystagmus or strabismus. She demonstrated a positive horizontal oculocephalic response. She showed hypertonia in her extremities and trunk muscles, aggravated by tactile stimulation. Flexor contractures were present in her extremities, and she made involuntary, purposeless movements with her hands. The Babinski sign was positive bilaterally.

Brain MRI indicated moderate pontine hypoplasia and significant cerebellar hypoplasia, while the corpus callosum remained normally sized (Figure 1A). The bilateral cerebellar hemispheres were affected asymmetrically (Figure 1B). Three-dimensional (3D) reconstruction from bone computed tomography (CT) revealed a singular curve with right convexity at the thoracolumbar junction (Figure 1C).

**Figure 1:**
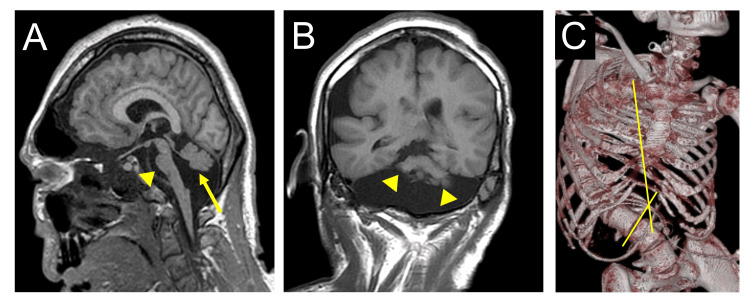
Brain MRI and bone CT findings in patient 1 (A) The T1-weighted sagittal brain MRI image shows microcephaly, intact corpus callosum, mild pontine hypoplasia (arrowhead), and significant vermis hypoplasia (arrow). (B) The T1-weighted coronal brain MRI image shows asymmetrically hypoplastic cerebellar hemispheres with a proportionally reduced vermis (arrowheads). (C) Three-dimensional reconstruction from a bone CT scan reveals a singular curve with right convexity at the thoracolumbar junction.

When she was transferred to our institution at age 38 years, she was receiving zonisamide 300 mg/day, clobazam 9.5 mg/day, phenytoin 115 mg/day, and perampanel 7 mg/day. After admission, no clinical seizures were observed, and electroencephalography showed no epileptiform discharges, including spikes or sharp waves (Figure 2). Therefore, zonisamide, clobazam, and phenytoin were discontinued, lacosamide 200 mg/day was introduced, and the perampanel dose was reduced to 4 mg/day. We managed to wean her off the ventilator during the day. Nonetheless, her generalized muscle hypertonia, scoliosis, and joint contractures in her extremities progressively deteriorated. Due to repeated vomiting after gastrostomy tube feedings, we transitioned to using a trans-gastric jejunal tube for feeding, which proceeded without trouble.

**Figure 2:**
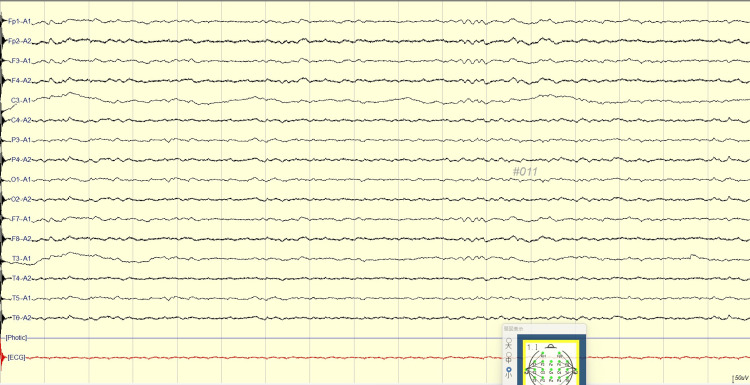
Electroencephalography findings in patient 1 after admission to our institution No epileptiform discharges, including spikes or sharp waves, are observed.

Patient 2

Patient 2, a 31-year-old woman, was born to non-consanguineous parents. Her birth length was unremarkable, with a weight of 3166 g and a head circumference of 31 cm. Diagnosed with an atrial septal defect and a ventricular septal defect in the heart seven days post-birth, she struggled with eye tracking and sound localization by eight months. Although she could roll over, she could not sit independently. By 10 months, she managed to crawl but soon developed an infantile spasm, escalating to intractable epilepsy unresponsive to various antiepileptic drugs. Her motor functions progressively declined, leading to a bedridden status and nasogastric tube feeding at two and a half years old due to recurrent aspiration pneumonia. Admitted to our institution at the age of 29, measured 121 cm in height, weighed 24 kg, and had a head circumference of 40 cm.

Her facial features included thick eyebrows, a broad nasal bridge, and large ears (Figure 3A), along with severe scoliosis and overriding toes. On neurological examination, she was alert but remained nonverbal and unresponsive to commands. Although unable to track visually, she could orient to sound sources. Loud noises elicited a startle reflex affecting her whole body, yet she exhibited neither nystagmus nor strabismus. A horizontal oculocephalic response could not be elicited. She could no longer roll over but could lift her head while lying on her back. Her limbs were contracted, yet within their limited range of motion, her limb muscles appeared normotonic. Conversely, her truncal muscles showed hypotonia. The upper limbs engaged in involuntary, aimless movements. Bilateral plantar reflexes were flexor. Brain MRI indicated moderate pontine hypoplasia and severe hypoplasia of the cerebellum, with the corpus callosum size remaining normal (Figure 3B). The bilateral cerebellar hemispheres were asymmetrically affected (Figure 3C). The 3D bone CT revealed a single thoracic curve with right convexity (Figure 3D).

**Figure 3:**
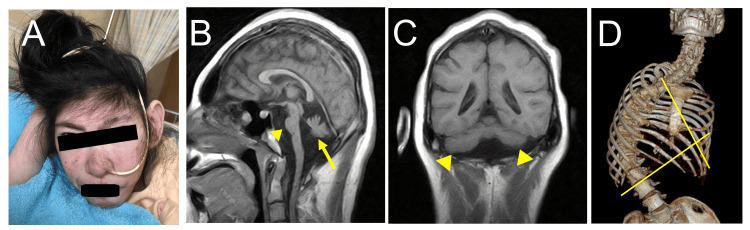
Clinical findings in patient 2 (A) The face with microcephaly, thick eyebrows, a broad nasal bridge, and large ears. (B) The T1-weighted sagittal brain MRI image shows microcephaly, intact corpus callosum, mild pontine hypoplasia (arrowhead), and significant vermis hypoplasia (arrow). (C) The T1-weighted coronal brain MRI image shows asymmetrically hypoplastic cerebellar hemispheres with a proportionally reduced vermis (arrowheads). (D) Three-dimensional reconstruction from a bone CT scan reveals a single thoracic curve with right convexity.

Despite nasogastric tube feeding, she frequently vomited and suffered from pneumonia, prompting a switch to nasojejunal tube feeding with a pump, which was successfully achieved. Before admission to our institution, she was treated with multiple antiseizure medications, including nitrazepam 5 mg/day, zonisamide 180 mg/day, gabapentin 200 mg/day, sodium valproate 500 mg/day, and levetiracetam 1750 mg/day. Despite these treatments, she continued to experience one to two tonic seizures daily and 5 to 10 myoclonic seizures per day. After admission, her antiseizure medications were gradually adjusted. At the present age of 31 years, she is receiving sodium valproate 500 mg/day, zonisamide 180 mg/day, perampanel 6 mg/day, and lacosamide 200 mg/day. Although seizure frequency improved, brief tonic seizures still occur weekly.

Patient 3

Patient 3, a 50-year-old woman, was born to non-consanguineous parents. Her birth followed a normal pregnancy and delivery. She weighed 2780 g, measured 47 cm in length, and had a head circumference of 31 cm. From the outset, she struggled with feeding issues, including poor sucking. Diagnosed with microcephaly at four months, she managed to hold her head up by six months. By 12 months, she could sit and begin bottom-shuffling, progressing to standing with assistance at 2 years old. At age 6, she could walk with support. Although she vocalized, she did not develop significant verbal skills. By age 27, she lost the ability to walk. At 48, she measured 140 cm in height, weighed 33 kg, and had a head circumference of 44 cm. She exhibited facial dysmorphisms, including a broad nasal bridge and large ears, as well as mild scoliosis and overriding toes.

On neurological examination, she was alert but remained nonverbal and unable to follow commands. She displayed restrictions in the upward and downward movements of her left eye without nystagmus. A horizontal oculocephalic response could not be elicited. Although she was unable to track visually, she could orient herself to sounds. She could roll over and sit unsupported, but standing remained impossible even with assistance. Her limbs showed contractures, yet her limb muscles appeared normotonic within their range of motion. Conversely, her truncal muscles showed hypotonia. The upper limbs engaged in involuntary, non-purposeful movements. Bilateral plantar reflexes were flexor. Brain MRI showed mild pontine hypoplasia with sparing of pontine bulging, and significant cerebellar hypoplasia, with the corpus callosum maintaining normal size (Figure 4A). The cerebellar hemispheres were asymmetrically affected (Figure 4B). The 3D bone CT scan revealed a single thoracic curve with right convexity (Figure 4C). Despite being on a dysphagia diet, she occasionally experienced aspiration. She was prescribed two antiepileptic drugs, levetiracetam and topiramate, with tonic seizures occurring only once every few years. She managed to eat a texture-modified diet orally.

**Figure 4:**
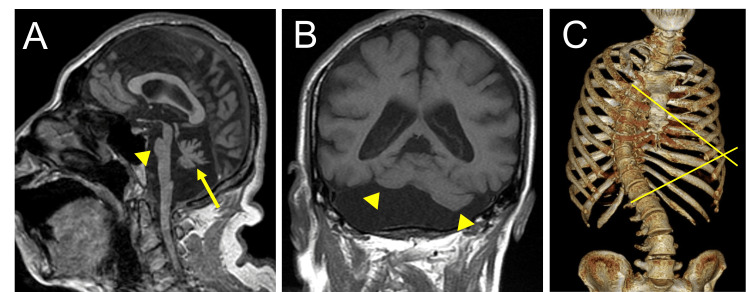
Brain MRI and bone CT findings in patient 3 (A) The T1-weighted sagittal brain MRI image shows microcephaly, intact corpus callosum, mild pontine hypoplasia (arrowhead), and significant vermis hypoplasia (arrow). (B) The T1-weighted coronal brain MRI image shows asymmetrically hypoplastic cerebellar hemispheres with a proportionally reduced vermis (arrowheads). (C) Three-dimensional reconstruction from a bone CT scan reveals a single thoracic curve with right convexity.

Based on the history, symptoms, and MRI findings of the three patients, we suspected MICPCH. To investigate the *CASK* gene, implicated in MICPCH, we employed the following methods. We extracted genomic DNA from the patients' peripheral blood using standard techniques. We then amplified all coding exons and exon-intron boundaries of the *CASK* gene by multiplex polymerase chain reaction (PCR) and sequenced them using a next-generation sequencer (NGS) (NextSeq 2000; Illumina Inc., USA). For identifying pathogenic variants, we compared the sequences against the human genome reference GRCh38/hg38 (Kazusa DNA Research Institute, Kisarazu, Japan). Subsequently, we purified mRNA according to the manufacturer's protocols (Nippon Gene, Japan) and synthesized cDNA using the Clone Avian Myeloblastosis Virus First-Strand cDNA Synthesis Kit (Invitrogen, USA). We amplified the targeted region in exon 17 of the *CASK* gene for patient 1 and in exon 12 for patient 2 by PCR and cloned them using the Zero Blunt PCR Cloning Kit (Invitrogen, USA). Sanger sequencing of multiple clones confirmed the variants identified by NGS. For patient 3, in whom NGS indicated a copy-number variant (CNV) in the exons of the *CASK* gene, we conducted Multiplex Ligation-dependent Probe Amplification (MLPA) analysis. Then, as with patients 1 and 2, we amplified the targeted region, including the CNV, cloned it, and performed Sanger sequencing.

Gene analysis revealed that patient 1 had a heterozygous nonsense variant, NM_003688.3: c.1609C>T, p.(Arg537*), in the coding region of exon 17 of the *CASK* gene (Figure 5A), previously reported as pathogenic/likely pathogenic in ClinVar. Patient 2 exhibited a heterozygous frameshift variant due to an 11-bp deletion, NM_003688.3: c.1079_1089del, p.(Leu360Glnfs*2), in the coding region of exon 12 of the *CASK* gene (Figure 5B). Because we did not analyze the *CASK* genes of her parents, we could not determine whether the variant was de novo. The variant is predicted to introduce a premature termination codon in exon 12 of *CASK* and is expected to result in nonsense-mediated mRNA decay. To our knowledge, this variant has not previously been reported in the literature or public databases, including gnomAD, ClinVar, iJGVD, and HGMD. According to the American College of Medical Genetics and Genomics/Association for Molecular Pathology (ACMG/AMP) guidelines, the variant was classified as likely pathogenic based on PVS1 (predicted loss of function due to a frameshift variant in a gene where loss of function is a known disease mechanism) and PM2_supporting (absence from population databases) [9]. In patient 3, the multiplex ligation-dependent probe amplification (MLPA) analysis identified a heterozygous duplication of exons 12-14 of the *CASK* gene (Figure 5C). Sanger sequencing confirmed a tandem duplication of exons 12-14 (Figure 5D). To our knowledge, this intragenic duplication has not been reported in the literature or public databases. The duplication is predicted to disrupt the reading frame and introduce a premature termination codon 24 amino acids downstream from the start of the duplicated exon 12, resulting in truncation of the CASK protein. Therefore, this CNV was considered likely pathogenic because it is predicted to cause loss of function in CASK, for which loss of function is an established disease mechanism in MICPCH.

**Figure 5:**
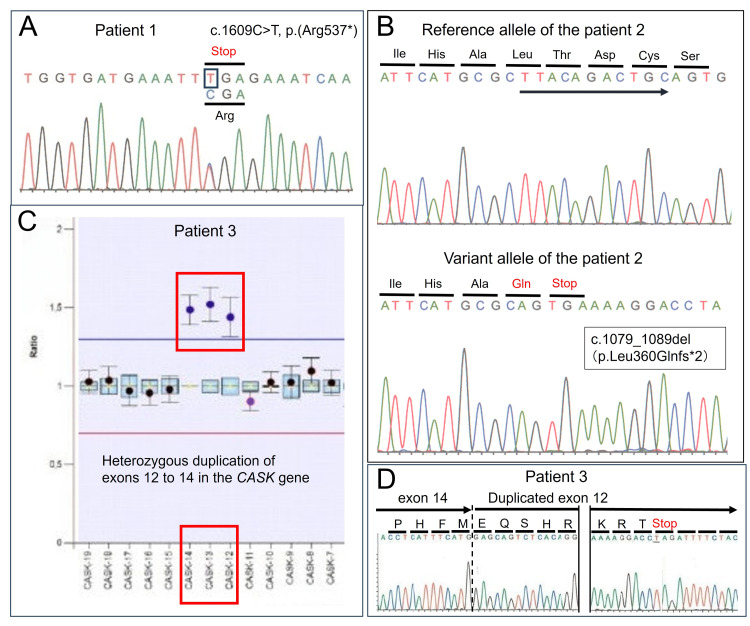
Genetic findings in the three patients with MICPCH associated with CASK aberrations MICPCH: Intellectual disability and microcephaly with pontine and cerebellar hypoplasia, CASK: Calcium/calmodulin-dependent serine protein kinase (A) Sequencing analysis of patient 1 showing a heterozygous nonsense variant in exon 17 of the *CASK* gene (c.1609C>T, p.(Arg537*)) resulting in a premature stop codon. (B) Sequencing analysis of patient 2 showing a heterozygous 11-bp deletion in exon 12 of the *CASK* gene (c.1079_1089del, p.(Leu360Glnfs*2)), resulting in a frameshift and premature stop codon 2 amino acids downstream of the altered codon. (C) Multiplex ligation-dependent probe amplification analysis of patient 3 showing a heterozygous duplication of exons 12–14 in the *CASK* gene (red boxes). (D) Sequencing analysis of patient 3 confirms a tandem duplication of exons 12–14. The duplicated exon 12 introduces a premature stop codon 24 amino acids downstream from the start of the duplicated exon 12. The dashed vertical line indicates an exon–exon boundary.

## Discussion

We found two crucial clinical points. First, in adult patients with MICPCH, we highlight the diversity of the *CASK* gene aberrations and the heterogeneity in clinical severity. To date, researchers have reported nearly 200 cases of MICPCH having *CASK* gene aberrations [7]. There was no mutational hot spot in the *CASK* gene. In females, most *CASK* gene aberrations associated with MICPCH are loss-of-function variants [7]. In our cases, patient 1 exhibited a nonsense variant, patient 2 a frameshift caused by an 11-bp deletion, and patient 3 a CNV with exon 12-14 duplication that introduces an early stop codon. Therefore, the *CASK* gene aberrations in all three patients represent loss-of-function variants. Regardless of the diversity of *CASK* aberrations in MICPCH, the core phenotype is remarkably similar among female patients. In particular, brain MRI demonstrates consistent findings that pontine and cerebellar hypoplasia with a normal-size corpus callosum [2,6]. This finding can be a diagnostic hallmark of MICPCH. Our patients also had common core phenotypes comprising severe developmental delay/intellectual disability, severe postnatal microcephaly, growth retardation, scoliosis, and similar MRI findings. The phenotypic similarity among female patients with MICPCH is attributable to the fact that most *CASK* gene aberrations are loss-of-function variants. These lead to *CASK* null alleles through nonsense-mediated mRNA decay [2,4]. Conversely, symptomatic severity varies widely among previously reported MICPCH patients, independent of the specific variants [2,4]. Mild cases exist that can walk and speak a few words [2,4].

Brain MRI findings in MICPCH are characterized by proportional hypoplasia of the cerebellar hemispheres and vermis with a relatively preserved corpus callosum, often creating a “butterfly-like” appearance on coronal images [2,6]. Additional findings include varying degrees of pontine hypoplasia, enlarged fourth ventricles, and mild cortical simplification [2,6]. Previous studies have reported substantial variability in the severity of cerebellar and pontine hypoplasia, ranging from mild abnormalities to near absence of the cerebellar hemispheres [2,6]. Although the severity of vermis hypoplasia has been reported to partially correlate with developmental milestones such as ambulation and language acquisition, MRI findings alone do not always reliably predict functional or intellectual outcomes [2,6].

Interestingly, despite the marked differences in clinical severity among our patients, their MRI findings were relatively similar, particularly regarding pontine and cerebellar hypoplasia. Patients 1 and 2 showed severe functional impairment, severe scoliosis, and dependence on tube feeding, whereas patient 3 achieved assisted ambulation, had milder scoliosis, and retained oral intake. These findings suggest that factors beyond structural MRI abnormalities, including genetic or other modifying factors, may contribute substantially to phenotypic severity in MICPCH. Although we did not perform an X chromosome inactivation (XCI) assay in our patients, some researchers suggest that the degree of XCI skewing may affect symptom severity in MICPCH, as in conditions such as Rett syndrome and Fragile X syndrome [2,5,10].

The second crucial clinical point is that we clarified the natural history of MICPCH by investigating adult patients, thereby aiding the development of long-term management strategies. To date, most reported MICPCH cases involve children, with only a few reports of adults, the oldest being a 32-year-old patient [8]. Therefore, the clinical spectrum and long-term outcomes in adult patients with MICPCH remain underexplored. Our cases, particularly patients 1 and 3, exceed the previously reported age range for MICPCH. Because of advances in healthcare, individuals with developmental disabilities, such as Down syndrome, have a longer life expectancy than ever before [11]. Our cases suggested that individuals with MICPCH can also achieve extended life expectancy with adequate respiratory and nutritional management.

On the other hand, scoliosis emerged as a significant clinical challenge in our patients, particularly in patients 1 and 2, where it was severe and, to a lesser extent, in patient 3. Scoliosis may have contributed to our patients being bedridden or repeatedly vomiting under tube feeding with a stomach tube. Several studies have addressed scoliosis in patients with MICPCH [2,4,6]. Patients with scoliosis are generally around 10 years old, suggesting that scoliosis does not manifest in early childhood in MICPCH [2,4,6]. Our patient 1 exhibited exceptional hypertonia of the trunk muscles, whereas patients with MICPCH, including our patients 2 and 3, generally exhibit hypotonia of the trunk muscles [2,4,6]. In any case, abnormal truncal muscle tone may influence the development and progression of scoliosis.

The impact of scoliosis on daily living and quality of life was profound among our patients, underscoring the need for early orthopedic and physical therapy interventions to mitigate progression. Early and accurate diagnosis of MICPCH is paramount, as evidenced by the need for timely scoliosis detection and initiation of appropriate interventions. The advancement of genetic diagnostics holds profound significance for adults with severe intellectual and motor disabilities, such as MICPCH. Through genetic testing in adult patients, we can gain a deeper understanding of the disease's natural history, enabling future-oriented management plans and therapies [12]. A limitation of this case series is that some neonatal clinical information relied on parental recollection because the original medical records were unavailable due to the retrospective nature of these adult cases.

## Conclusions

Our adult cases expand the known age spectrum and phenotypic variability of MICPCH associated with *CASK* aberrations. Despite relatively similar MRI findings, the patients exhibited substantial differences in functional severity, epilepsy burden, and scoliosis progression, highlighting the dissociation between radiological and clinical severity in MICPCH. Our findings also emphasize scoliosis and long-term epilepsy management as important challenges in adult patients and support the value of early diagnosis and multidisciplinary long-term care.
